# Long-term Weight Loss as a Predictor of Mortality in Hemodialysis Patients

**DOI:** 10.2188/jea.JE20210389

**Published:** 2023-08-05

**Authors:** Takuhiro Moromizato, Ryoto Sakaniwa, Takamasa Miyauchi, Ryuhei So, Hiroyasu Iso, Kunitoshi Iseki

**Affiliations:** 1 Renal and Rheumatology Division, Internal Medicine Department, Okinawa Prefectural Nanbu Medical Center and Children’s Medical Center, Shimajiri, Okinawa, Japan; 2 Public Health, Department of Social Medicine, Osaka University Graduate School of Medicine, Osaka, Japan; 3 Division of Nephrology and Hypertension, Department of Internal Medicine, St Marianna School of Medicine, Kawasaki, Japan; 4 Okayama Psychiatric Medical Center, Okayama, Japan; 5 Clinical Research Support Center, Nakamura Clinic, Urasoe, Okinawa, Japan

**Keywords:** body mass index, mortality, haemodialysis, follow-up study

## Abstract

**Background:**

Serial weight decrease can be a prognostic predictor in chronic hemodialysis (HD) patients. We investigated the impact of long-term post-HD body weight (BW) changes on all-cause mortality among HD patients.

**Methods:**

This longitudinal cohort study and post-hoc analysis evaluated participants of a previous randomized controlled trial conducted between 2006 and 2011 who were followed up until 2018. Weight change slopes were generated with repeated measurements every 6 months during the trial for patients having ≥5 BW measurements. Participants were categorized into four groups based on quartiles of weight change slopes; the median weight changes per 6 months were −1.02 kg, −0.25 kg, +0.26 kg, and +0.86 kg for first, second, third, and fourth quartile, respectively. Cox proportional hazard regression was used to evaluate differences in subsequent survival among the four groups. BW trajectories were plotted with a backward time-scale and multilevel regression analysis to visualize the difference in BW trajectories between survivors and non-survivors.

**Results:**

Among the 461 patients, 404 were evaluated, and 168 (41.6%) died within a median follow-up period of 10.2 years. The Cox proportional hazard regression adjusted for covariates and baseline BW showed that a higher rate of weight loss was associated with higher mortality. The hazard ratios were 2.02 (95% confidence interval [CI], 1.28–3.20), 1.77 (95% CI, 1.10–2.85), 1.00 (reference), and 1.11 (95% CI, 0.67–1.83) for the first, second, third (reference), and fourth quartiles, respectively. BW trajectories revealed a significant decrease in BW in non-survivors.

**Conclusion:**

Weight loss elucidated via serial BW measurements every 6 months is significantly associated with higher mortality among HD patients.

## INTRODUCTION

Low body weight (BW) is a crucial clinical indicator and prognostic marker in chronic hemodialysis (HD) patients.^1^^,^^2^ Moreover, intradialytic and acute BW changes are critical targets to optimize fluid status.^3^ Meanwhile, long-term BW change is easily overlooked and rarely evaluated.^4^ However, long-term BW change can be a significant prognostic marker for HD patients because it may reflect the chronic influence of protein-energy wasting (PEW). PEW, characterized by decreased serum albumin or cholesterol levels; loss of body, adipose, or muscle mass; and inadequate protein or energy intake, predicts poor prognosis in dialysis patients.^5^ Lean body mass, which comprises the skeletal muscle and organs, adipose tissues, and bone, is chiefly associated with long-term BW change.^6^ Hence, long-term BW change may reflect the longitudinal impact of PEW, thus functioning as a clinically useful prognostic marker in HD patients.

The impact of long-term BW change over a 2–3-year time period on mortality, however, has been rarely evaluated.^4^^,^^7^ Accordingly, the significance of long-term BW change in HD patients remains unclear. Our previous study showed that a higher baseline BW was associated with lower mortality during the initial 3 years of follow-up, whereas it was associated with higher mortality between 3 and 10 years of follow-up.^8^ The opposite relationship between BW and mortality before and after 3 years implied that long-term change of BW, in addition to baseline BW, may affect mortality. Elucidating the impact of long-term BW change on mortality may help clarify the differential mechanisms for the opposite relationship.

We conducted a randomized controlled trial, the Olmesartan Clinical Trial in Okinawan Patients Under Okinawa Dialysis Study (OCTOPUS), on chronic HD patients with hypertension. The trial collected comprehensive and adjudicated baseline clinical information and repeated measurements of BW every 6 months for future analysis. The present study aimed to investigate the impact of long-term BW changes on all-cause mortality among chronic HD patients, using data from the OCTOPUS trial.

## METHODS

### Study design population

This was a longitudinal cohort study and post-hoc analyses of adjudicated baseline data, repeatedly measured weight data, and prognosis data of the OCTOPUS trial participants. The OCTOPUS study was conducted between June 2006 and June 2011.^9^^,^^10^ A detailed protocol of the OCTOPUS study was published previously.^10^ We followed up for the prognosis of the participants until July 31, 2018.

**Figure 1.**
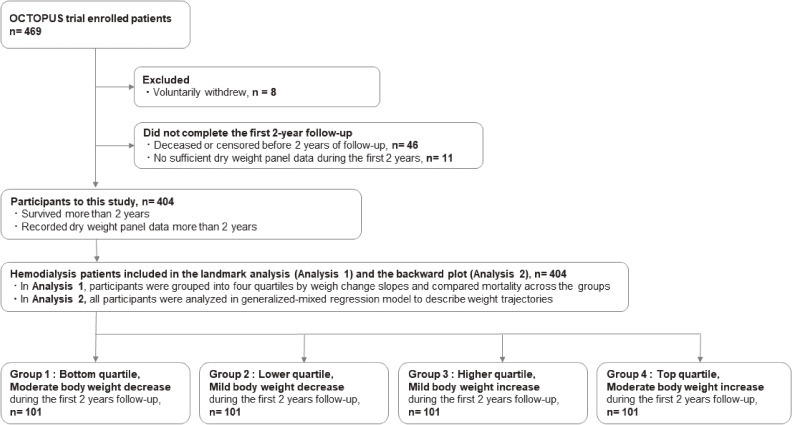
Flowchart for the study participants

The inclusion and exclusion criteria of this study were the same as those of the OCTOPUS trial. As for the inclusion criteria, participants had to be ambulatory patients undergoing HD three times a week, aged 20–79 years at enrolment, and have a blood pressure ≥140/90 mm Hg to be enrolled.^10^ Among the 469 original OCTOPUS participants, eight patients withdrew voluntarily, leaving 461 patients to be evaluated. To clarify the impact of long-term BW changes on mortality, we included 404 patients who had survived for more than 2 years and had at least five waves of BW data recorded during the trial (Figure 1).

This follow-up study was approved by the IRB committee of the Muribushi Project for Residency Training in Okinawa (IRB# 2018-1). All HD units that participated in the OCTOPUS trial displayed a written notification of the start of this investigation (since June 1, 2018) to ask the participants’ intentions of their withdrawal.

### Measurements of BW, body mass index, and covariates

Post-dialysis BW was recorded for a week every 6 months during the OCTOPUS trial, but not after the trial. Following the prespecified schedule provided by the OCTOPUS trial team, BW measurements were conducted at each dialysis session for a week directly by dialysis staff using a medical weighing scale and were reported as data for the trial. Titration of the scale and confirmation of BW with 2–3 assessments for each measurement were routinely performed. Body mass index (BMI) was calculated with the formula BW in kilograms divided by height in meters squared.

Regarding the definition of “long-term” for weight change, we adopted 2 years as the tentative timeframe to observe weight changes. In the study cohort, 5% of patients died every year.^8^ Therefore, a 3-year follow-up period would have reduced the sample size by 15% or increased the amount of missing data, both of which would affect the study’s statistical power and introduce selection bias. Additionally, a 1-year timeframe cannot distinguish between long-term BW change and acute BW change caused by hemodynamic change or infectious diseases; thus, our analysis excluded BW changes within 1 year.

Prespecified baseline covariates, demographics, past medical history, and laboratory data were collected at the initiation of the OCTOPUS trial following its protocol.^10^ The percentage of patients with at least one missing datum in baseline covariates among all participants was 10.1%; we employed complete-case analysis for multivariate adjustment.

### Definition of the primary outcome

Data on all-cause mortality were collected in the same manner both during and after the trial. Data on the cause of death were collected and adjudicated during the trial, whereas they were collected via chart review after the trial. Surviving participants were censored on the date of loss to follow-up, their kidney transplantation, or the predetermined censoring date of July 31, 2018.

### Statistical analysis

Categorical variables of exposures and outcomes were evaluated using frequency tables, whereas continuous variables were summarized and described graphically based on the exposure status. Two additional major analysis steps were planned to achieve our primary aim. These are described below.

#### Analysis 1: Landmark analysis^11^

A slope of post-HD BW change during the first 2 years of follow-up was calculated for each patient with a least square regression model. The participants were grouped into four groups based on quartiles of the BW change slope: from the bottom slope group (moderate BW decrease group) to the top slope group (moderate BW increase group) corresponding to the first to fourth quartiles, respectively (eFigure 1).

The mortality rates in the groups were compared using Kaplan–Meier curves. Moreover, Cox-regression hazard ratios without adjustment; adjusted for age and sex; and adjusted for multiple variables, such as age, sex, baseline post-dialysis BW, diabetes, history of myocardial infarction, history of stroke, prior hemodialysis duration, baseline pre-dialysis systolic blood pressure, serum albumin, serum phosphate, and albumin and phosphate interaction terms were calculated for each group.

#### Analysis 2: Trajectories of BW before endpoints during the OCTOPUS trial

The point estimates of BW and their standard errors were calculated every 6 months during the trial using time-scale multilevel regression models. For the multilevel regression models, backward time points were specified as a time variable, and the multilevel regression model was defined as a random intercept and slope model. Backward time points were described using a backward time-scale, such that month 0 was the month of death for deceased patients and month of censorship for alive patients during the trial. Thus, this model indicated that month 0 was the point for measuring the intercept in the analysis. Furthermore, the beta-coefficient was determined as the slope that revealed the difference in BW changes between non-survivors and survivors.^12^

The slope terms (time, time squared, and time cubed) allowed us to evaluate BW changes for 2–5 years prior to the endpoints, whereas BW changes of patients who ended follow-up within 2 years were not included in the analyses. The proportion of deceased and alive patients and BW values of the participants are listed in eTable 1. To assess whether longitudinal trends of BW differed between non-survivors and survivors, we introduced the following interaction terms: between death status and time, between death status and time squared, and between death status and time cubed. Hence, we examined whether the introduction of these interaction terms and baseline covariates could improve the model fitness (eTable 2). The endpoint status (intercept) of our final mixed model was adjusted for the following baseline covariates: age, sex, diabetes, smoking, and duration of dialysis before enrolment. Rate of change (slope) in our final mixed model was adjusted for the following interaction terms: between death status and time, between duration of dialysis before enrolment and time, between death status and time squared, and between death status and time cubed.

Using the backward time-scale multilevel regression models, we drew trajectory lines of BW based on survival status. As for sensitivity analyses, the trajectory lines of BW and BMI stratified by death status were generated separately for male and female patients (eFigure 2A, eFigure 2B, eFigure 2C, and eFigure 2D). In addition, the trajectory lines of BW stratified by death status were generated separately for patients with duration of dialysis before enrolment <5 years and for those with duration ≥5 years (eFigure 2E and eFigure 2F). All statistical analyses were performed using STATA 16.1 MP (Stata Corp., College Station, TX, USA). All *P*-values presented were two-tailed, and *P* < 0.05 was considered statistically significant.

## RESULTS

### Patient characteristics

The mean patient age was 59.1 years, and 61% of the patients were men. A total of 168 patients (41.6%) died within a median follow-up of 10.2 years. The follow-up rate of this study was 98.3%. The baseline characteristics of the 404 patients (101 patients per group) included in analysis 1 are shown in Table 1. The mean rate of BW change per 6 months was −1.02 kg in Group 1, −0.25 kg in Group 2, +0.26 kg in Group 3 (reference group), and +0.81 kg in Group 4. Baseline height, BW, and BMI were not significantly different among the four groups. Group 4 showed the highest prevalence of diabetes history (43%) and the shortest length of HD (35 months on average), whereas the other three groups showed similar values. Group 1 showed the lowest rate of diuretic use. Other variables, except antihypertensive drugs, were identical among these four groups.

**Table 1.** Baseline characteristics accordings to groups of body weight change

	All patients		Group 1 (Moderate BW decrease)		Group 2 (Mild BW decrease)		Group 3 (Mild BW increase)		Group 4 (Moderate BW increase)		*P*-value	
	*n* = 404		*n* = 101		*n* = 101		*n* = 101		*n* = 101			
**Rate of body weight change (kg/6 months)**	−0.01 (−0.56 to 0.43)		−1.02 (−1.38 to −0.74)		−0.25 (−0.37 to −0.16)		0.26 (0.10–0.33)		0.81 (0.59–1.09)			
**Rate of body mass index change (kg/m^2^/6 months)**	0.00 (−0.22 to 0.17)		−0.43 (−0.57 to −0.30)		−0.11 (−0.15 to −0.06)		0.10 (0.04–0.13)		0.32 (0.23–0.43)			
**Baseline information about body components**												
Height (cm)	158.2	(9.0)	156.9	(8.3)	157.2	(9.5)	158.9	(8.3)	159.6	(9.6)	0.09	
Dry weight (kg)	57.3	(11.0)	57.8	(9.9)	55.3	(10.3)	57.6	(10.2)	58.7	(13.0)	0.16	
BMI (kg/m^2^)	24.0	(3.7)	24.6	(3.7)	23.4	(3.6)	23.8	(3.4)	24.0	(4.1)	0.19	

**Demography**												
Men (%)	61		61		56		67		58		0.41	
Age, years (years)	59.1	(11.7)	60.8	(11.5)	59.5	(11.5)	58.2	(11.2)	58.0	(12.7)	0.30	
**Baseline Medical history**												
Medical history												
Stroke (%)	15		12		19		11		18		0.27	
Myocardial infarction (%)	4		3		5		3		3		0.83	
Diabetes (%)	30		30		24		25		43		0.01	^*^
Smoking												
Ex-smoker (%)	19		24		18		17		19		0.40	
Current (%)	18		13		19		25		16			

**Hemodialysis (HD) associated conditions**												
Prior HD duration (months)	64.5 (27–126)		77.0 (38–141)		81.0 (36–132)		71.0 (31–138)		35.0 (14–80)		<0.01	^*^
Primary renal disease											0.44	
Chronic glomerulonephritis (%)	45		49		44		41		45			
Diabetic nephropathy (%)	31		29		25		27		43			
IgA nephropathy (%)	5		4		5		8		3			
Nephrosclerosis (%)	4		3		5		6		3			
Pre-HD systolic BP (mm Hg)	161.7	(17.5)	161.9	(17.8)	162.3	(18.4)	161.7	(16.0)	160.8	(18.0)	0.94	
Pre-HD pulse (bpm)	78.2	(11.3)	76.7	(12.9)	77.4	(10.7)	79.4	(12.1)	79.3	(9.3)	0.30	
Post-dialtysy CTR (%)	50.2	(4.8)	51.1	(4.5)	50.6	(4.8)	50.0	(4.6)	49.3	(5.3)	0.07	

**Pre-HD laboratory values**												
Albumin (g/L)	38.0	(3.1)	37.0	(3.2)	38.0	(2.9)	38.0	(3.0)	38.0	(3.3)	0.31	
Hemoglobin (g/dL)	10.4	(1.3)	10.4	(1.4)	10.4	(1.1)	10.6	(1.2)	10.4	(1.3)	0.55	
Sodium (mmol/L)	138.3	(3.2)	138.1	(3.6)	138.4	(2.7)	138.2	(3.3)	138.4	(3.1)	0.84	
Potassium (mmol/L)	4.8	(0.7)	4.8	(0.7)	4.8	(0.7)	4.8	(0.7)	4.7	(0.7)	0.88	
Phosphorus (mmol/L)	1.9	(0.5)	1.9	(0.5)	1.8	(0.4)	1.9	(0.5)	1.9	(0.5)	0.53	

ARB, angiotensin II receptor blocker; BMI, body mass index; BP, blood pressure; BW, body weight; CTR, cardio-thoracic ratio; HD, hemodialysis.

^a^Values are a percentage, mean (standard deviation), or median (inter-quatile range). Parametric values were written as mean values (standard deviations), and parametric values among four groups were compared with ANOVA-tests. Non-parametric values were writtens as median values (inter-quartile range), and non-parametric values among four groups were compared with Kruskal Wallis tests. Frequencies among four groups were copared with chi-square tests.

^b *^P-value <0.05.

### Results of the landmark study

**Figure 2.**
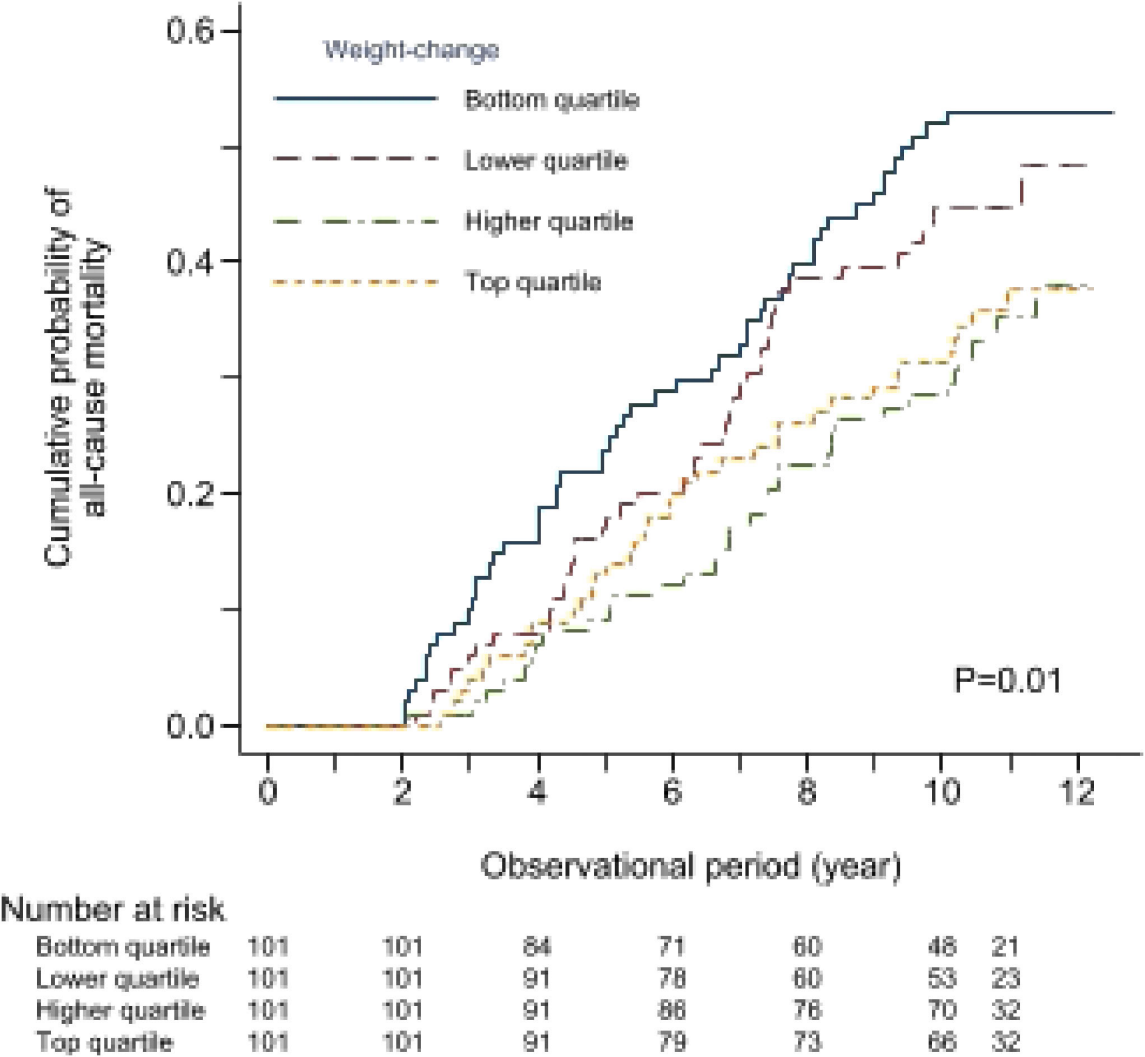
All-cause mortality according to quartiles of weight change

Figure 2 shows the Kaplan–Meier curve in the four groups stratified by slopes of BW change. These Kaplan–Meier survival curves were plotted after the first 2 years of follow-up as the landmark analysis. The moderate decrease in BW (Group 1) was associated with the highest mortality risk, and a mild increase in BW (Group 3) was associated with the lowest mortality risk. The *P-value* for the generalized log-rank test was 0.015.

Table 2 shows the comparison of outcome values among the four groups. Univariate, age/sex-adjusted, and multivariate-adjusted Cox hazard ratios of mortality increased as the rate of BW loss increased. The hazard ratios adjusted for multiple variables, including baseline post-dialysis BW, were 2.02 (95% confidence interval [CI], 1.28–3.20), 1.77 (95% CI, 1.10–2.85), 1.00 (reference), and 1.11 (95% CI, 0.67–1.83) for the first, second, third (reference), and fourth quartiles, respectively. These values were similar to hazard ratios that were not adjusted for baseline post-dialysis BW. Moreover, Table 2 describes the cause of death among the four groups. In all groups, the leading cause of death was infectious disease.

**Table 2.** Comparisons of survival outcome indexes according to quartiles of body weight change in chronic hemodialysis patients

	Group 1 (Moderate BW decrease)	Group 2 (Mild BW decrease)	Group 3 (Mild BW increase)	Group 4 (Moderate BW increase)	*P*-value
Person-years	834.1	870.4	960.3	936.5	
No. at risk	101	101	101	101	
No. of death	53	45	34	36	0.012^#^

Univariate HR (95%, CI)	1.88 (1.22–2.89)^**^	1.51 (0.97–2.35)	1.00 (Reference)	1.10 (0.69–1.76)	
Age-sex adjusted HR (95%, CI)	1.84 (1.20–2.83)^**^	1.54 (0.98–2.40)	1.00 (Reference)	1.10 (0.69–1.76)	
Multivariate adjusted HR (95%, CI)^b^	2.02 (1.28–3.20)^**^	1.77 (1.10–2.85)^*^	1.00 (Reference)	1.11 (0.67–1.83)	

Causes of death (%)					0.419^$^

Cardiovascular disease	13	19	10	13	
Infectious disease	21	12	13	14	
Cancer	5	4	6	1	
Other/Unknown	14	9	5	8	

BW, body weight; CI, confidence interval; HR, hazard-ratio; No, number.

^a^Values are a person-years, number, HR effect sizes (95%, CI), or percentage (%).

^b^Multivariate Cox proportional hazard regression model adjusted for following variables: age, gender, baseline post-dialysis body weight, diabetes, history of myocardial infarction, history of stroke, prior hemodialysis duration, baseline pre-dialysis systolic blood pressure, serum albumin, serum phosphate, albumin and phosphate interaction term. Group 3 was set as a reference group to measure hazard ratios. Since serum albumin data had 41 missing values, the number of analyzed patients was 363 in the multivariate Cox proportional hazard regression model. The number of total deceased patients in the model was 158.

^c *^P < 0.05, ^**^P < 0.01; ^#^*P*-values for comparing frequencies of the number of deaths are calculated by chi-squared test; ^$^*P*-values for comparing frequencies of causes of deaths are calculated by Fisher’s exact test.

### Time-mixed trajectories of BW and BMI before endpoints

Figure 3A and Figure 3B show the difference in the BW trajectory lines between non-survivors and survivors during the trial and the difference in the trajectory lines of BMI between the two groups, respectively. A persistent decrease in BW and BMI was observed in non-survivors of both sexes regardless of prior HD duration (eFigure 2A, eFigure 2B, eFigure 2C, eFigure 2D, and eFigure 2F). Further, in both sexes, the difference in BW and BMI between non-survivors and survivors became significant at 2 years before the endpoints. The number of non-survivors and survivors, actual BW and BMI values, and estimated BW and BMI values at each time point are listed in eTable 1.

**Figure 3A.**
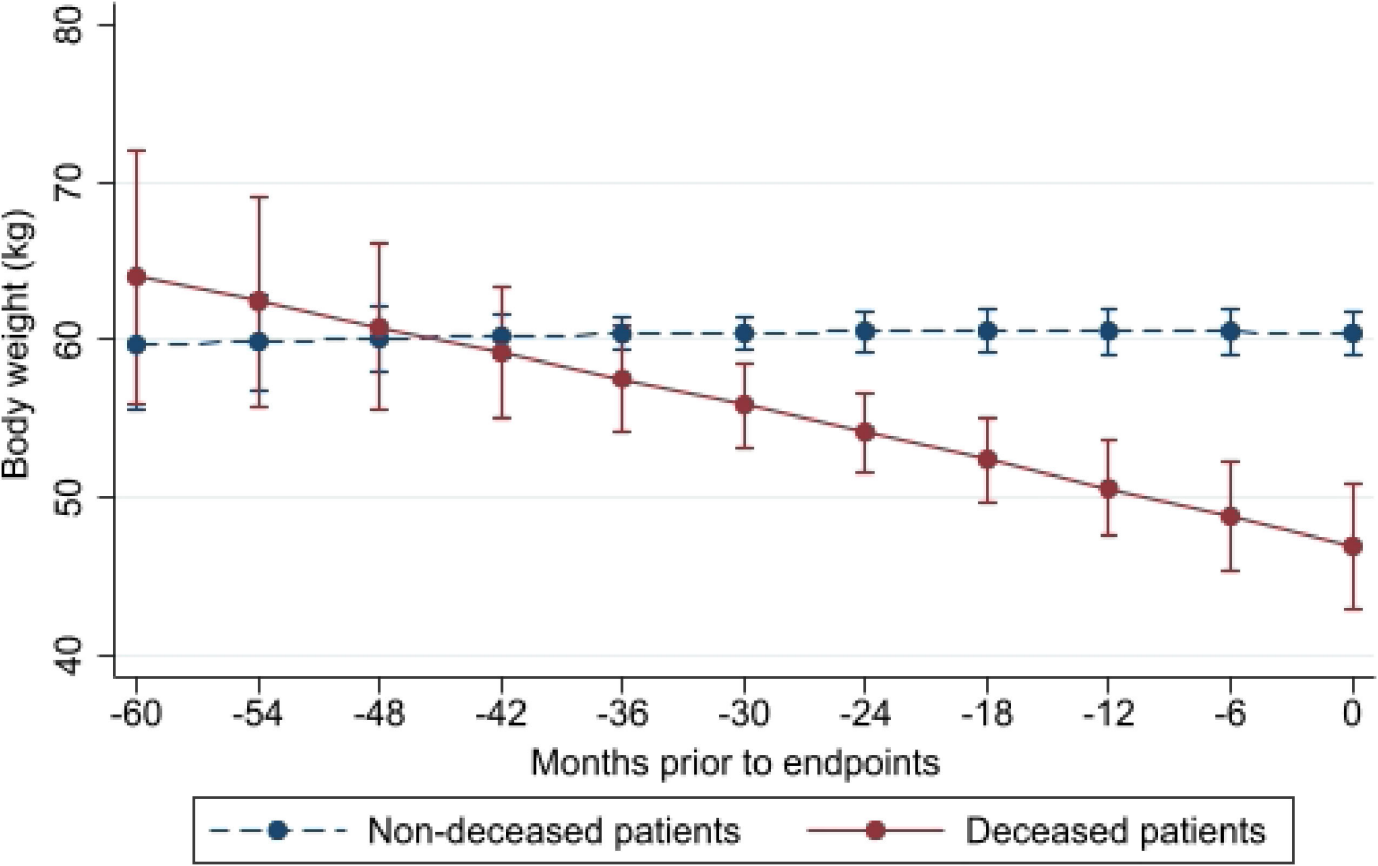
Means and standard errors of body weight at each backward point of measurement from death or survival endpoints in deceased and non-deceased patients: Difference in body weight trajectory, *P* < 0.0001

**Figure 3B.**
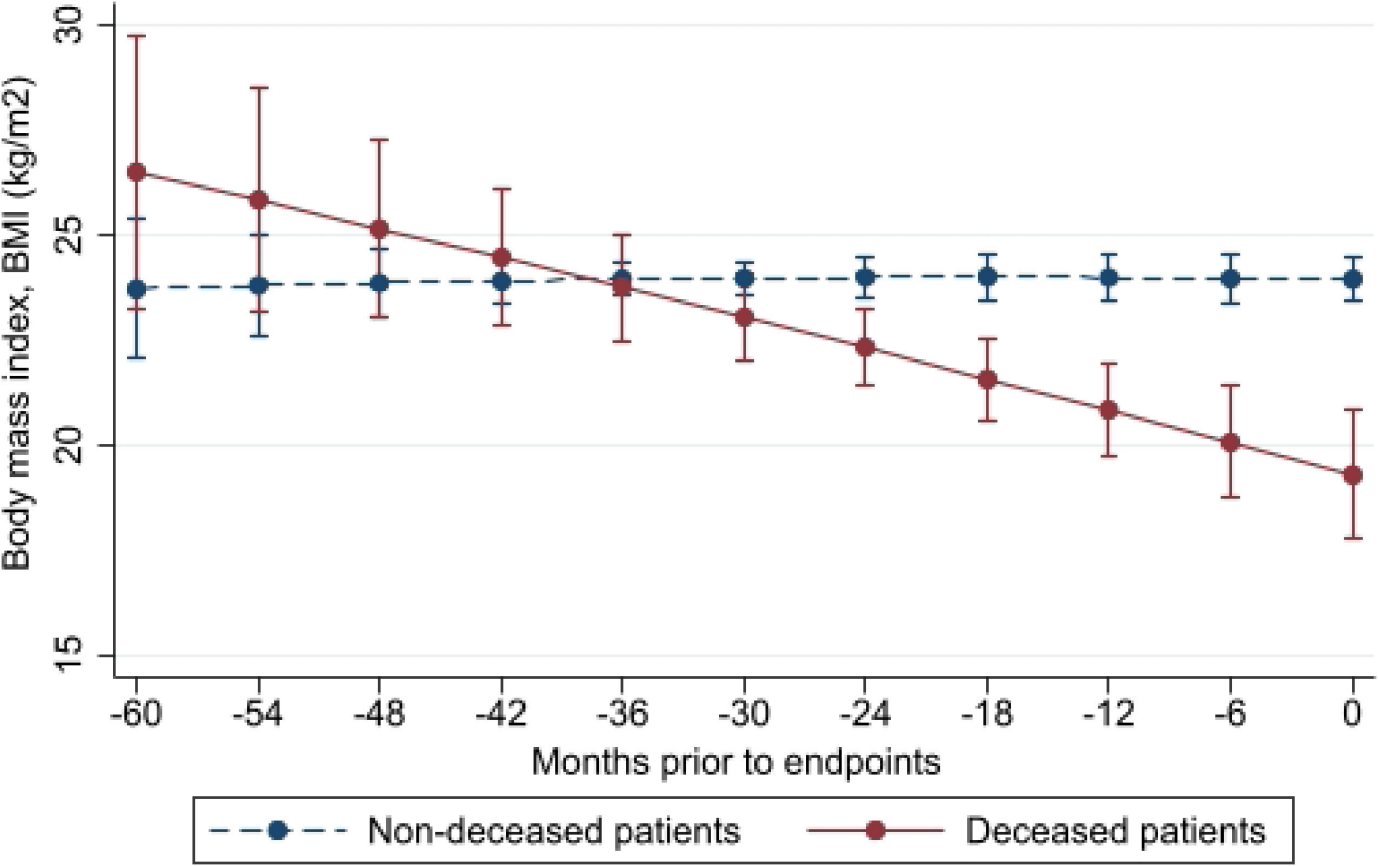
Means and standard errors of body mass index (BMI) at each backward point of measurement from death or survival endpoints in deceased and non-deceased patients: Difference in BMI trajectory, *P* < 0.0001

## DISCUSSION

The effect of long-term BW changes on the mortality risk in HD patients remains unclear. Using landmark analysis, we found that a higher rate of BW loss was associated with a higher mortality risk in these patients. The trajectory lines showed that non-survivors showed persistent BW loss before their deaths and that survivors showed a stable BW trajectory.

Moreover, the statistical impacts of long-term BW changes on mortality were elucidated. Compared to patients with a stable or minimal increase in BW, those with a BW loss of 1.0 kg in 6 months had twice the risk of all-cause mortality. Further, trajectory BW lines showed that in both sexes, the difference in BW and BMI changes started at 2 years before the endpoints.

Although clinically intuitive, the association between a higher rate of BW loss and higher mortality risk in maintenance HD patients has been rarely investigated due to the shortage of sufficient serial BW data.^4^ The impact of baseline BW on their outcomes has been chiefly investigated, instead of BW changes. Few studies have evaluated the association between BW change and mortality among chronic HD patients.^7^^,^^13^^–^^15^ Further, the previous studies have primarily aimed to clarify the impact of obesity on the outcome, as evidenced by the high prevalence of obese patients in those studies.^14^^,^^16^

Moreover, the slope of BW in these previous studies was generated using a maximum of 6–12 months data, which may not be long enough to exclude the impact of acute disease. To the best of our knowledge, our study was the first to investigate the trend in BW change for >2 years and its association with mortality risk in chronic HD patients. In addition, trajectory lines helped visualize the time discrepancy of BW impact on mortality. We can recognize that not only baseline BW at a certain time point but also weight change over a period of time impacts mortality; thus, serial weight data collected preferably over a period of 2 years would be helpful to determine the impact of BW on all-cause mortality.

Baseline body components of HD patients have been heavily investigated.^17^^–^^19^ HD patients have a lower BMI than the matched general population.^1^^,^^20^ The suggested mechanisms of the high prevalence of lower BMI in HD patients were their low energy intake, increased energy expenditure, increased catabolism, and muscle wasting.^21^ Importantly, low baseline BW and BMI are proven predictors of poor prognosis in HD patients.^2^^,^^22^

A limited number of studies captured long-term weight changes as prognosis predictors in maintenance HD patients, and their timeframe of weight change was generally 6–12 months.^7^^,^^13^^,^^15^ Notably, fluctuations in seasonal BW change were reported to be small (around 0.5 kg) in the Japanese dialysis population.^23^

Although the association between long-term BW change and mortality in HD patients seems clinically intuitive, the underlying mechanisms of this relationship remain to be elucidated. The association may be a consequence of three prevalent and intercorrelated conditions in HD patients: PEW, sarcopenia, and loss of energy preservation. The prevalence of PEW was 40–60% in HD patients, and PEW prevailed at the stage of chronic kidney disease (CKD) stage 3b.^24^^,^^25^ However, the criterion for BW loss in CKD-related PEW (>10% of BW over 6 months) is too strict to be applied in our study. The current results clarify the impact of relatively small changes in BW (1.7% of baseline BW) over 6 months. A structural review of 44 studies on CKD-related PEW and cachexia suggested the necessity of milder BW change criteria (<5% over 6 months) because BW monitoring depending on current BW and BMI change cut-off may cause late diagnosis and intervention.^26^

Regarding the cause of death as an outcome measure for assessing the association between BW change and mortality, patients with lower BMI were at a higher risk of death from non-cardiovascular diseases, including infectious diseases and wasting.^27^ Similarly, patients with higher BW loss in our study had a higher risk of death from infectious diseases and unknown causes, consistent with the findings of previous studies.^28^^,^^29^

Possible interventions for improving mortality by preventing BW weight loss included the prevention of PEW conditions, sarcopenia, and loss of energy preservation.^21^^,^^30^ Adequate control of inflammation, appropriate protein and energy intake, and proper dialysis prescription are crucial to suppress causative factors of PEW, including uremia, oxidative stress, and hyper-catabolism. Additionally, strategies for increasing physical activity are critical to alleviate PEW consequences and to avoid sarcopenia.^25^ However, long-term benefits of exercise in dialysis patients have not been consistently observed in previous studies.^30^ Further, improving the efficacy of exercise is a crucial treatment target in dialysis patients.^30^

We were concerned about the specificity of the association between BW loss and mortality in HD patients because weight reduction was observed 9 years before death in the general population. However, the weight reduction rate was much higher in our HD patients (1.02 kg over 6 months) than that in the general population (−0.39 kg over 1 year).^31^

This study had invaluable strengths. Time-dependent changes were recorded via repeated measurements of post-dialysis weight every 6 months in a sufficiently large number of patients. Moreover, the association between weight change and mortality was evaluated and visualized simultaneously from the aspect of exposure (landmark analysis) and outcome analysis with a time-mixed model with a backward time-scale. To our knowledge, the association between long-term weight change and mortality in the HD population has not been evaluated using trajectory visualization of long-term weight changes. The visualization method may advance our understanding of the background mechanism of the time discrepancy of BW impact on mortality, which is generally observed in obese HD patients.^1^ Although the discrepancy was observed in relatively non-obese HD participants of our study, the means of baseline BMI at the enrolment were 23.0 (standard deviation, 3.7) kg/m^2^ in deceased patients and 22.7 (standard deviation, 3.4) kg/m^2^ in surviving patients.^8^ Further, comprehensive baseline data and a high follow-up rate (98.3%) allowed us to observe less biased associations.

However, several limitations need to be considered when interpreting our results. First, this study could not distinguish between unintentional and intentional weight loss. However, we assumed that almost all weight changes were unintentional, chiefly because most of the patients were not obese. Furthermore, BW was usually monitored and optimized by dialysis staff. Second, unmeasured variables, such as serial data of nutritional indicators (eg, normalized protein catabolic rate), direct body compositions, and dialysis efficiencies, hindered us from speculating prevention strategies for long-term BW change. Third, the findings were influenced by the observational nature of the study, as this study relied on a post-hoc analysis that introduced additional follow-up. The outcome variable after the OCTOPUS trial was not prespecified and adjudicated. Participants were from a restricted population of chronic HD patients with hypertension in Japan, and 50% of them received angiotensin II receptor blocker as an intervention in the OCTOPUS trial. Moreover, our study included only HD patients, the inclusion of peritoneal dialysis patients might allow us to further inspect mechanisms for the association between BW variation and mortality among CKD patients.

In conclusion, repeated evaluations of weight change every 6 months allowed us to capture long-term weight loss that predicts a higher mortality risk in HD patients. Future studies referring to our results should clarify prevention strategies for weight loss to improve survival of HD patients.

## SUPPLEMENTARY MATERIAL

eFigure 1. Slope of weight change (kg) by 6 months

eFigure 2A. Means and standard errors of body weight at each backward point of measurement from death or survival endpoints in deceased and non-deceased male patients: Difference in body-weight trajectory, *P* < 0.0001

eFigure 2B. Means and standard errors of body weight at each backward point of measurement from death or survival endpoints in deceased and non-deceased female patients: Difference in body-weight trajectory, *P* < 0.0001

eFigure 2C. Means and standard errors of body mass index (BMI) at each backward point of measurement from death or survival endpoints in deceased and non-deceased male patients: Difference in BMI trajectory, *P* < 0.0001

eFigure 2D. Means and standard errors of body mass index (BMI) at each backward point of measurement from death or survival endpoints in deceased and non-deceased female patients: Difference in BMI trajectory, *P* < 0.0001

eFigure 2E. Means and standard errors of body weight at each backward point of measurement from death or survival endpoints in deceased and non-deceased patients with prior haemodialysis (HD) duration <5 years (n = 192): Difference in body-weight trajectory, *P* < 0.0001

eFigure 2F. Means and standard errors of body weight at each backward point of measurem`ent from death or survival endpoints in deceased and non-deceased patients with prior haemodialysis (HD) duration ≥5 years (n = 212): Difference in body-weight trajectory, *P* < 0.0001

eTable 1. Estimated means of body weight, and body mass index of chronic hemodialysis patients at each backward point of measurement from death or survival endpoints during the OCTOPUS trial

eTable 2. Time-scale mixed model development steps by adding level 1 variable (time), level 2 variables, and polynomial terms
