# Supplementary material for: Long-term Weight Loss as a Predictor of Mortality in Hemodialysis Patients
Source: J Epidemiol. 2023 Aug 5;33(8):390–7. doi: 10.2188/jea.JE20210389 (PMC10319526; doi:10.2188/jea.JE20210389)

eTable 1. Estimated means of body weight, and body mass index of chronic hemodialysis patients at each backward point of measurement from death or survival endpoints during the OCTOPUS trial

	Points of measurements (months)										
**All patients**	**-60**	**-54**	**-48**	**-42**	**-36**	**-30**	**-24**	**-18**	**-12**	**-6**	**0**
**Number of patients who recorded body weight at each point of measurement**											
Patients deceased during the OCTOPUS trial	2	12	19	31	46	53	59	59	59	59	24
Patients survived during the OCTOPUS trial	38	119	191	302	345	344	345	345	343	340	151
Total patients during the OCTOPUS trial	40	131	210	333	391	397	404	404	402	399	175
**Actual mean body weight (SD)**											
Patients deceased during the OCTOPUS trial	56.2 (16.0)	51.4 (7.5)	53.9 (8.9)	53.6 (8.1)	54.6 (9.9)	54.9 (9.7)	54.1 (10.0)	53.8 (9.9)	53.2 (10.0)	52.1 (9.8)	50.6 (10.4)
Patients survived during the OCTOPUS trial	61.7 (11.8)	59.6 (10.9)	60.5 (11.6)	60.7 (11.7)	60.3 (11.7)	60.5 (12.0)	60.3 (12.0)	60.6 (12.2)	60.3 (12.2)	60.3 (12.3)	59.2 (12.9)
**Estimated mean body weight (SE)**											
Patients deceased during the OCTOPUS trial	64.0 (4.1)	62.4 (3.4)	60.8 (2.7)	59.2 (2.1)	57.6 (1.7)	55.9 (1.4)	54.1 (1.3)	52.4 (1.4)	50.6 (1.6)	48.8 (1.8)	46.9 (2.1)
Patients survived during the OCTOPUS trial	59.7 (2.1)	59.9 (1.5)	60.1 (1.1)	60.2 (0.7)	60.4 (0.5)	60.5 (0.5)	60.5 (0.6)	60.6 (0.7)	60.6 (0.8)	60.5 (0.8)	60.5 (0.7)
**Estimated mean BMI (SE)**											
Patients deceased during the OCTOPUS trial	26.5 (1.7)	25.9 (1.4)	25.2 (1.1)	24.5 (0.8)	23.8 (0.6)	23.0 (0.5)	22.3 (0.5)	21.6 (0.5)	20.8 (0.6)	20.1 (0.7)	19.3 (0.8)
Patients survived during the OCTOPUS trial	23.7 (0.8)	23.8 (0.6)	23.9 (0.4)	23.9 (0.3)	23.9 (0.2)	24.0 (0.2)	24.0 (0.2)	24.0 (0.3)	24.0 (0.3)	24.0 (0.3)	23.9 (0.3)

BMI, Body mass index; OCTOPUS, Olmesartan clinical trial in Okinawan patients under OKIDS; OKIDS, Okinawa Dialysis Study; SD, Standard deviation; SE, Standard error.

[a)] Values are counts of patients, mean (SD), and estimated means (SE). Estimated mean values were all calculated with time-scale mixed multivariable regression for the following baselinie covariates: age, gender, diabetes, smoking, prior hemodialysis duration, linear time term, quardratic time term, and cubic time term.

eTable 2. Time-scale mixed model development steps by adding level 1 variable (time), level 2 variables, and polynomial terms

		Model A	Model B	Model C	Model D	Model E	Model F	Model G
**Fixed effects**								
**Endpoint status (kg)**								
	Intercept	59.447 ***	59.049 ***	62.796 ***	72.391 ***	71.822 ***	72.008 ***	71.991 ***
	Death			-8.765 ***	-6.739 ***	-6.784 ***	-7.115 ***	-7.603 ***
	Length of past HD			-0.343 ***	-0.270 **	-0.257 **	-0.252 **	-0.248 **
	Age				-0.288 ***	-0.288 ***	-0.293 ***	-0.292 ***
	Gender				10.024 ***	10.620 ***	10.606 ***	10.635 ***
	Diabetes				3.428 **	3.838 **	3.636 **	3.621 **
	Smoking				-1.352	-1.370	-1.422	-1.394
**Rate of change (linear term, kg per months)**								
	Intercept		-0.017 ***	0.016	0.117 ***	0.103 ***	0.089 **	0.087 *
	Death			-0.106 ***	-0.094 ***	-0.095 ***	-0.162 ***	-0.286 ***
	Length of past HD			-0.003 ***	-0.003 ***	-0.003 ***	-0.003 **	-0.002 **
	Age				-0.001 ***	-0.001 **	-0.001 **	-0.001 **
	Gender				-0.015			
	Diabetes				-0.010			
	Smoking				0.000			
**Rate of change (quardratic term and cubic term)**								
	$Months^2$ (quadratic term)						0.000	0.000
	Death × $Months^2$ (quadratic term)						-0.002 *	-0.008 **
	$Months^3$ (cubic term)							0.000
	Death × $Months^3$(cubic term)							0.000 *
**Variance components**								
**Level 1**	Within person	5.46 ***	2.930 ***	2.925 ***	2.925 ***	2.925 ***	2.260 ***	1.896 ***
**Level 2**	Variance in endpoint status	137.33 ***	158.342 ***	143.725 ***	109.105 ***	109.202 ***	107.864 ***	105.820 ***
	Variance in							
	Linear term		0.010 ***	0.009 ***	0.009 ***	0.009 ***	0.046 ***	0.108 ***
	Quadrative term						0.000 **	0.000 **
	Cubic term							0.000 **
	Covariance in							
	Linear term with endpoint status		0.557 ***	0.410 ***	0.390 ***	0.392 ***	0.425 ***	0.352 ***
	Quadrative term with endpoint status						0.000 **	-0.003
	Cubic term with endpoint status							0.000
	Linear term with Quadrative term						0.001 **	0.004 **
	Linear term with Cubic term							0.000 *
	Quadrative term with Cubic term							0.000 *
**Goodness of fit**								
	Degree of freedom	3	6	10	18	15	20	26
	Wald statistics	.	9.05	83.67	250.8	248.0	245.8	255.9
	Deviance statistics	17048.8	15995.3	15927.1	15789.7	15792.1	15540.2	15386.7
	AIC	17054.8	16963.3	15947.1	15825.7	15822.1	15580.2	15438.8
	BIC	17073.1	17000.2	16008.1	15935.5	15913.5	15702.1	15597.3

AIC, Akaike's information criteria; BIC, Bayesian information criteria; HD, Hemodialysis.

[a)] **Model A** is the unconditional mean model; **Model B** is the unconditional growth model; **Model C** is the mixed model that a death status and a length of prior HD duration were added as predictors of slope and intercept term in the Model B; **Model D** is the mixed model that between person variables (age, gender, diabetes, smoking) were included as predictors of slope and intercept terms of the Model C; **Model E** is the mixed mode that three between person variables (gender, diabetes, smoking) were removed because of non significance as predictors of slope terms in the Model E; **Model F** is the mixed model that quadratic term of time variable (months) was added to both intercept and slope terms of the Model E; **Model G** is the mixed model that cubic tem of time variable (months) was added to the Model F. Our final model is the model G. The number of analyzed patients for all models were 404.

[b)] * p <0.05, ** p<0.01, *** p<0.001.

eFigure 1. Slope of weight change (kg) by 6 months

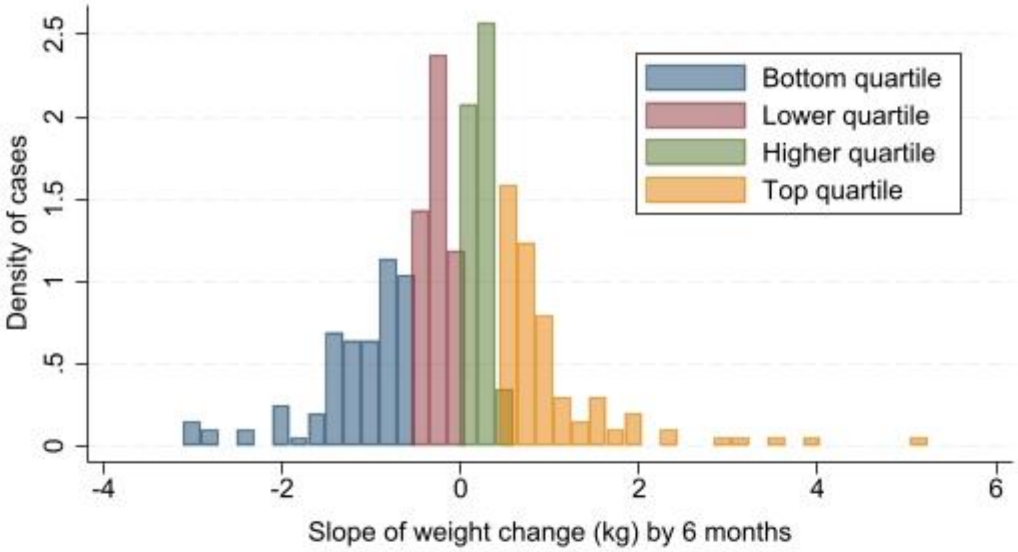

eFigure 2A. Means and standard errors of body weight at each backward point of measurement from death or survival endpoints in deceased and non-deceased male patients: Difference in body-weight trajectory, *P*<0.0001

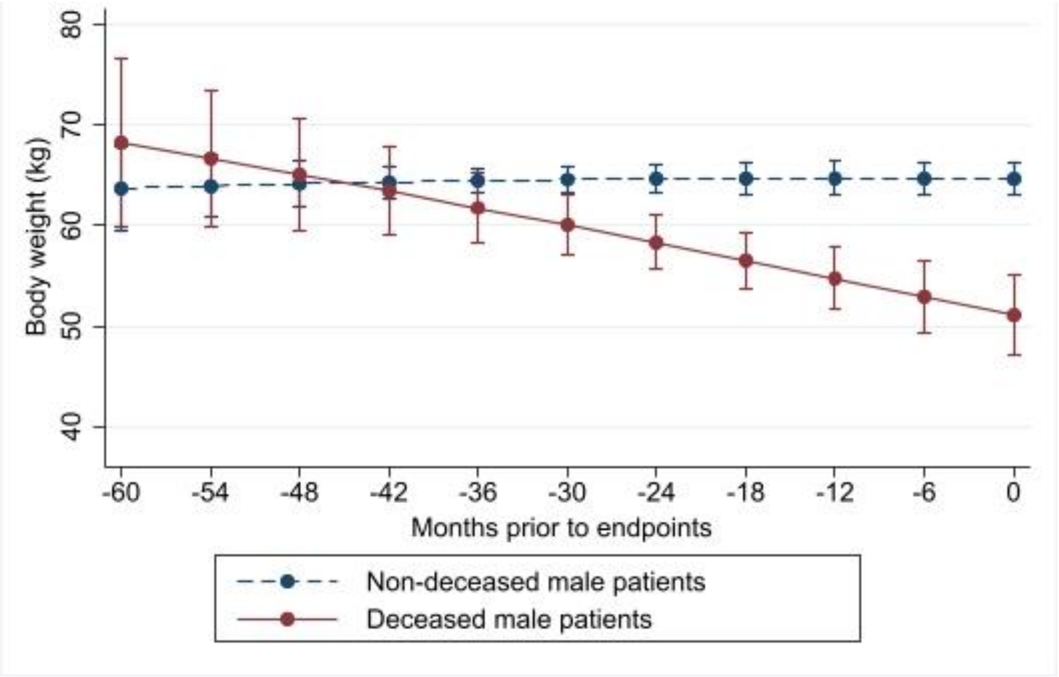

eFigure 2B. Means and standard errors of body weight at each backward point of measurement from death or survival endpoints in deceased and non-deceased female patients: Difference in body-weight trajectory, *P*<0.0001

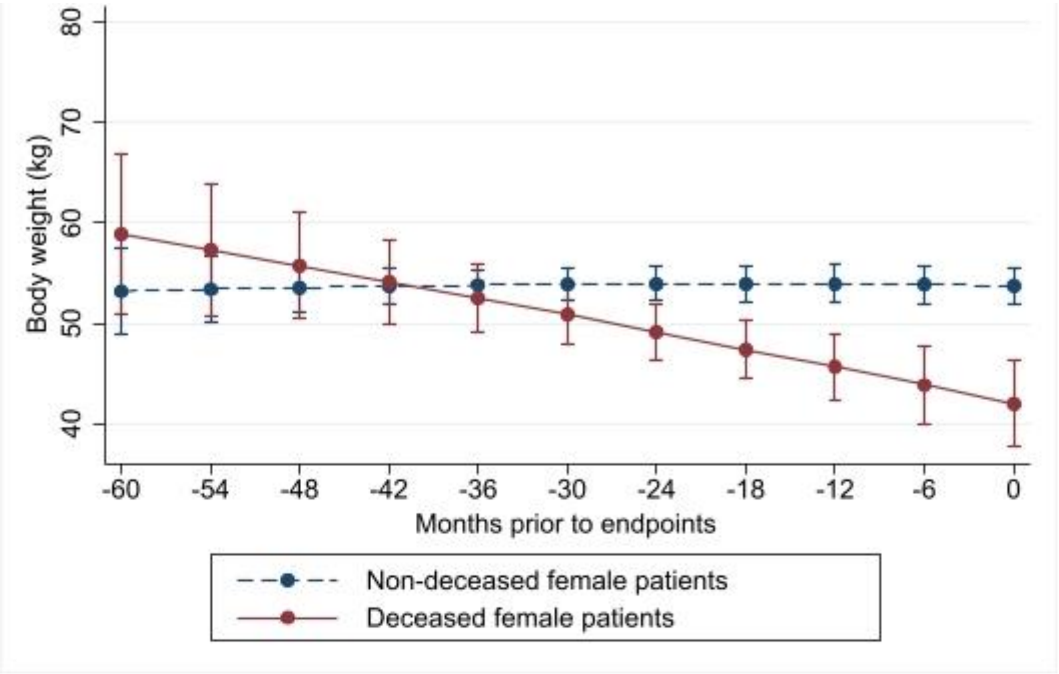

eFigure 2C. Means and standard errors of body mass index (BMI) at each backward point of measurement from death or survival endpoints in deceased and non-deceased male patients: Difference in BMI trajectory, *P*<0.0001

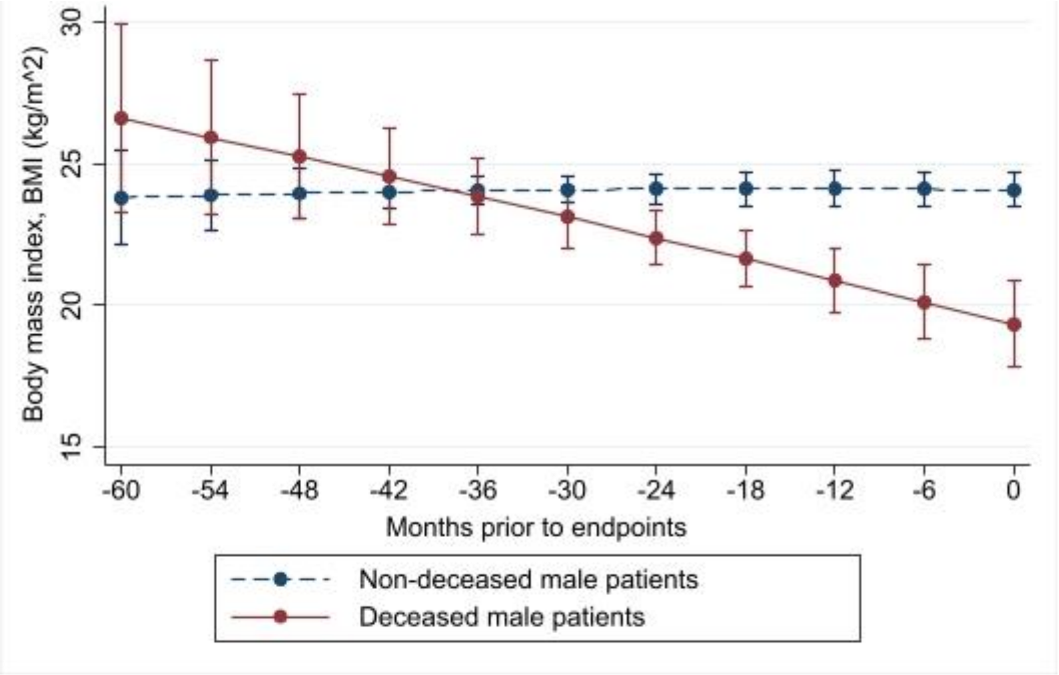

eFigure 2D. Means and standard errors of body mass index (BMI) at each backward point of measurement from death or survival endpoints in deceased and non-deceased female patients: Difference in BMI trajectory, *P*<0.0001

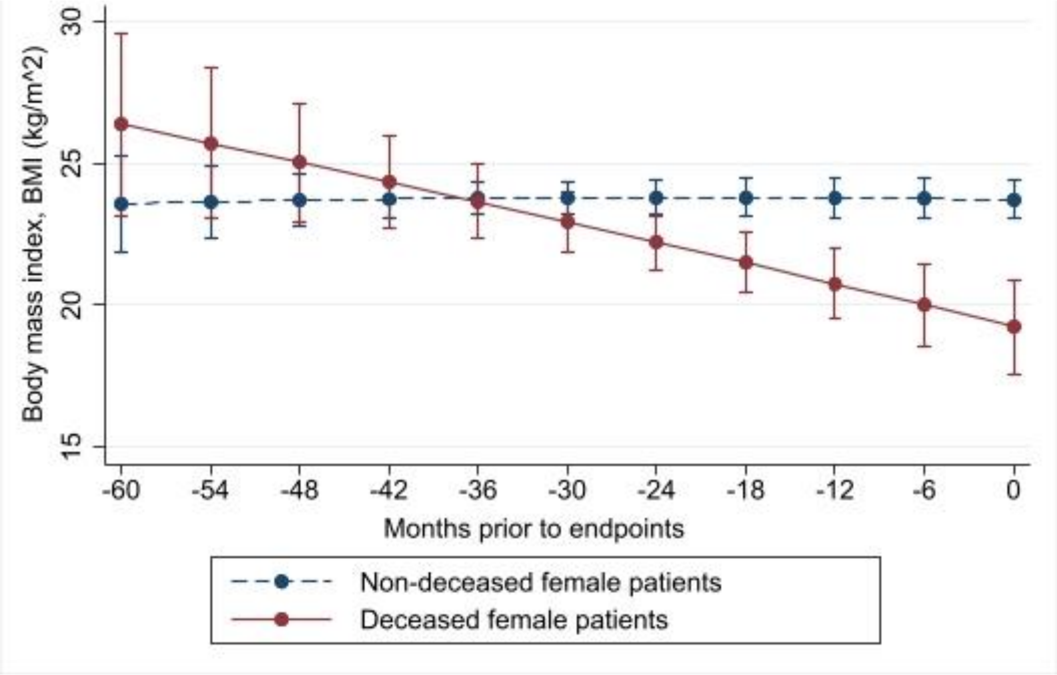

eFigure 2E. Means and standard errors of body weight at each backward point of measurement from death or survival endpoints in deceased and non-deceased patients with prior haemodialysis (HD) duration <5 years (n=192): Difference in body-weight trajectory, *P*<0.0001

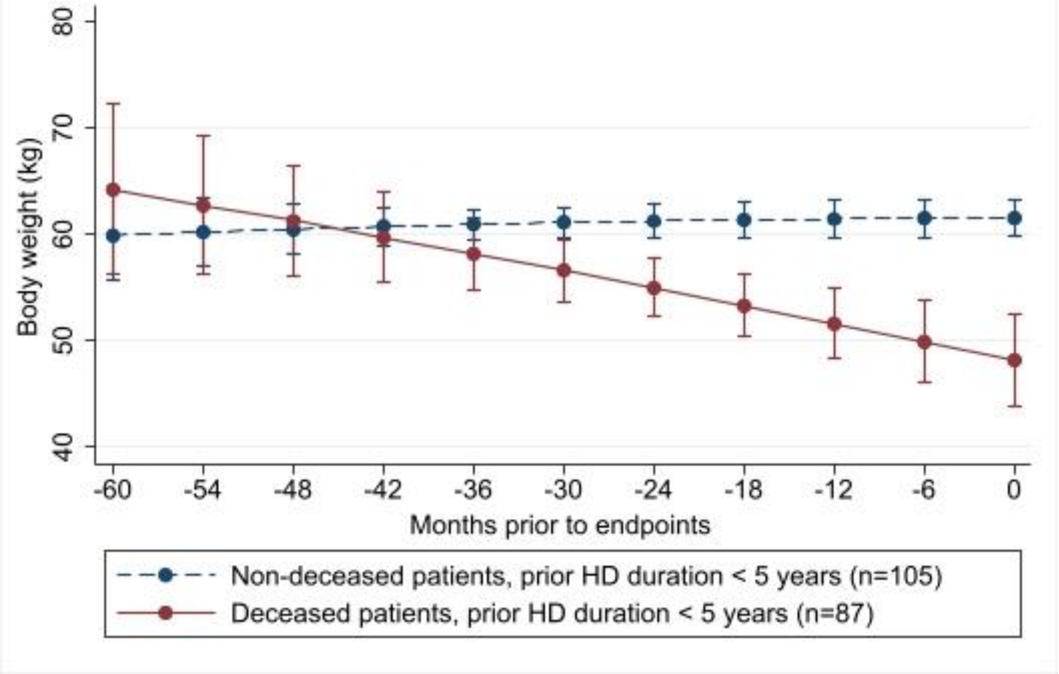

eFigure 2F. Means and standard errors of body weight at each backward point of measurem`ent from death or survival endpoints in deceased and non-deceased patients with prior haemodialysis (HD) duration ≥5 years (n=212): Difference in body-weight trajectory, *P*<0.0001

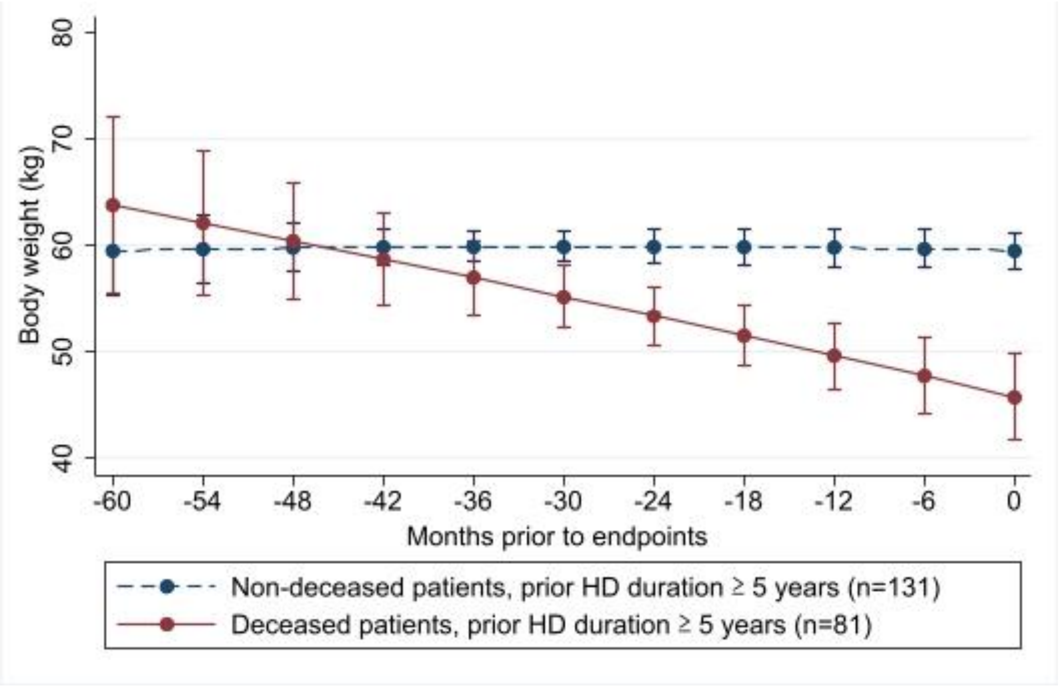

Supplement: Supplementary file 1 [file je-33-390-s001.pdf]
